# Review on the occurrence of the *mcr-1* gene causing colistin resistance in cow's milk and dairy products

**DOI:** 10.1016/j.heliyon.2021.e06800

**Published:** 2021-04-16

**Authors:** Ágnes Nagy, Rita Székelyhidi, Erika Hanczné Lakatos, Viktória Kapcsándi

**Affiliations:** Department of Food Science, Faculty of Agricultural and Food Sciences, Szechenyi Istvan University, 9200, Mosonmagyaróvár, Lucsony utca 15-17, Hungary

**Keywords:** Mastitis, *mcr-1*, Colistin, Antibiotic resistance, ESBL, Cattle, Cheese, *E.coli*, *Enterobacteriaceae*

## Abstract

Both livestock farmers and the clinic use significant amount of antibiotics worldwide, in many cases the same kind. Antibiotic resistance is not a new phenomenon, however, it is a matter of concern that resistance genes (*mcr* - Mobilized Colistin Resistance - genes) that render last-resort drugs (Colistin) ineffective, have already evolved. Nowadays, there is a significant consumption of milk and dairy products, which, if not treated properly, can contain bacteria (mainly Gram-negative bacteria). We collected articles and reviews in which Gram-negative bacteria carrying the *mcr-1* gene have been detected in milk, dairy products, or cattle. Reports have shown that although the incidence is still low, unfortunately the gene has been detected in some dairy products on almost every continent. In the interest of our health, the use of colistin in livestock farming must be banned as soon as possible, and new treatments should be applied so that we can continue to have a chance in fighting multidrug-resistant bacteria in human medicine.

## 1 Introduction

### 1.1 Milk

Cow's milk is a nutritious liquid and known to be consumed by humankind for centuries. Its consumption had probably started with the domestication of the cow. Recent statistics show that milk consumption in the world is constantly increasing. The largest consumer of milk is India (77.7M tons), followed by the EU (33.4 M tons), and the next is the US (Shahbandeh 2020a). At least 3% of the US population consumes raw milk (Liu et al., 2020a). The major milk producers are also these three countries, with the EU leading the list (155M tons), followed by the US (99M tons) (Shahbandeh 2020b).

Singh et al. (2015) have shown that milk consumption increases with age and those in better social status consume more milk than their poorer counterparts. In addition, women consume slightly more milk than men. Because milk consumption is significant, the quality of the milk content is important. Mastitis is one of the most important and costly worldwide infectious diseases in the dairy industry. Mastitis parenchymatosa can be caused by several strains of bacteria, however, inflammations caused by *Escherichia coli* may be the most severe (Hamann 2010) Intramammary infections often recur and are easily transmitted (Döpfer et al. 1999, 2000). Plenty of antibiotics are used to treat and prevent infection (Timofte et al., 2014).

### 1.2 Colistin

Colistin (Yang et al., 2020; Li et al., 2005) has been used in the clinic since 1959, however, due to its nephrotoxicity and neurotoxicity, it has been replaced by other antibiotics since the 1970s. However, in the last 10 years, multidrug-resistant (MDR) pathogens have reappeared, which was traced back to clinical use (Silva et al., 2013).

Colistin is currently used as a last resort against MDR bacteria in clinical practice. It has been widely used in agricultural production and veterinary medicine for decades to treat infections caused by MDR bacteria in livestock, primarily for the treatment of intestinal diseases. It has been shown that a significant proportion of colistin and other polymyxins, which are much more widely used in animal husbandry, are transferred to humans through food. Intestinal colonizers readily exchange genetic material with any member of the microbiota (whether pathogenic or symbiont), which significantly increases the risk of infection (Stecher et al., 2012). A significant problem is that antibiotics are used not only to treat disease (Alcantara et al., 2020), but also as a prophylaxis (Catry et al., 2015) or even as a growth promoter (Vidovic and Vidovic, 2020). The review by Kempf et al. (2016) also describes that colistin is used in farm animals on several continents, particularly for the treatment of gastrointestinal disorders, and also as a growth promoter.

Saini et al. (2012) measured the frequency of colistin use in dairy farms in Canada, even though colistin is not officially licensed in veterinary medicine (Webb et al., 2017). The study describes that colistin is used very often and that antibiotic use increases in proportion to the amount of milk produced. In South Korea, from 2005 to 2015, on average 6–16 tons of colistin was given annually to food-producing animals (Belaynehe et al., 2018). In 2015, approximately 12,000 tons of colistin was used in food production, and the use of this antibiotic is estimated to increase to 16,500 tons by 2021 (Liu et al., 2016).

The inevitable consequence of the use of antibiotics is the development of bacterial resistance. The extent of this varies from bacteria to species and antibiotics, which is a rather complex process. Wang et al. (2017) tested 125 samples of livestock, poultry meat, milk and aquatic products in 2016, and showed that 40% of these samples had some antibiotic content.

In the case of Gram-negative bacteria, the range of antibiotics that can be administered has been narrowed significantly. There is a constant need for new antibiotics, but this situation cannot be sustained indefinitely and it is also costly. A more advisable approach would be to prevent the spread of resistance. It must be recognized that this is a global and geographically inseparable phenomenon, that certain MDR bacteria cause little or no medical problems and, last but not least, that bacterial resistance is a growing economic burden (Bacanli and Basaran, 2019). Perhaps the Swann report (Swann et al., 1969), as early as in 1969, was the first to seriously address the potential consequences of the overuse of antibiotics and drew attention to limiting them to prescription and to banning certain types. Following the publication of the report, Swann appeared before the English Parliament and sparked a serious debate between food manufacturers and health professionals. The spread of antibiotic resistance and resistance genes will only increase in the coming years (Zhao et al., 2020).

The use of colistin poses a serious risk, because resistance genes can be transferred from animals to humans (McEachran et al., 2015), and it is predicted that antimicrobial resistance will cause the death of 10 million people by 2050 (Luo et al., 2020).

### 1.3 *mcr-1* gene

Antimicrobial resistance (AMR) occurs when microorganisms change when exposed to an antimicrobial activity for an extended period of time or at very low concentrations. One of the leading health problems worldwide is AMR in human and veterinary medicine (Pardon et al., 2012). These microorganisms are sometimes called “superbugs”. As a result of the change, the drug used against them is no longer effective. This problem may be exacerbated if microorganisms become resistant to multiple antibiotics (like MDR). More and more attention is being paid to AMR transfer from animals to humans, especially when related to food production (Liebana et al., 2013; Madec and Haenni, 2018; Wegener 2003).

The *mcr-1* gene that causes resistance was discovered at the end of 2015, so research on this gene is quite new (Liu et al., 2016). It protects bacteria from colistin (also known as polymyxin E), which is a polypeptide antimicrobial agent. This gene encodes an enzyme called phosphatidylethanolamine transferase which transfers the phosphatidylethanolamine residue to the lipid A of the gram-negative bacterial cell membrane, to which colistin would bind (Guachalla et al., 2018). El-Sayed Ahmed et al. (2020) summarizes the characteristics of the mechanisms of colistin resistance. The primary mechanism of resistance to polymyxins is through modification of the bacterial outer membrane, which is essentially a chromosomally encoded resistance. In addition, it can develop through heteroresistance, where subpopulations of susceptible bacteria survive antibiotic levels above the minimum inhibitory concentrations (MIC). Because polymyxins have amphipathic nature, the efflux pump system may involved in the development of resistance. Colistin resistance follows primarily after exposure to colistin, but may develop without it. There are strains that have intrinsic colistin resistance, and in many cases the mechanism for the development of resistance is still unknown.

Shortly after the discovery of the *mcr-1* gene, it has been detected in almost all food-producing animal species in 57 countries, on 5 continents (Luo et al., 2020) (Skov and Monnet, 2016).

Most *mcr* genes were found in members of the family Enterobacteriaceae, and the prevalence was highest in *E.coli* (Elbediwi et al., 2019). However, the genes are also being detected in more and more species such as *Salmonella, Shigella sonnei, Klebsiella pneumoniae, Enterobacter aerogenes*, and *Enterobacter cloacae*. As early as in 2012 in a Nature article, Gilbert (2012) drew attention to the importance of antibiotic resistance. *mcr-1* was shown to alter the susceptibility of bacteria to unrelated antibiotics, which promotes the development of MDR (McGann et al., 2016; Sonnevend et al., 2016; Yang et al., 2017).

To date, nine *mcr*-family genes have been described (Hadjadj et al., 2019; Simoni et al., 2018; Xavier et al., 2016; Fukuda et al., 2018). Ling et al. (2020) summarized the geographical distribution of *mcr* genes. *mcr-1* and *mcr-9* genes have been detected on all continents. *mcr-4*, *mcr-6*, *mcr-7*, and *mcr-8* genes have only been found in a few countries, but have not yet been studied in many countries. Based on a phylogenetic study, *mcr-1* and *mcr-3* have the largest number of variants. And the most *mcr*-positive isolates are restricted to various Enterobacteriaceae species. Furthermore, 11 variants of the *mcr-1* gene have been found so far (AbuOun et al., 2017; Sun et al., 2018). Because the gene is located on a plasmid, it easily spreads to other bacteria (Hmede and Kassem 2019), thereby increasing the number of resistant strains, creating even MDR species. Phylogenetic analysis of *mcr-*1 genes isolated from *E. coli* was performed by Li et al. (2018). This study found that the strains acquired the gene independently, so clonal transmission is not the only pathway for the spread of the resistance gene. The gene has been detected in a large number of environmental samples (Anyanwu et al., 2020) (hospital source, contact surfaces at public transportation routes, water – lake, river, sea, public beach; aquaculture, soil, plant, sewage, wastewater, wildlife – birds, mammals, reptiles, fish and flies), manure ecosystem (pig slurry, farming soil), food (Ghafura et al., 2019) (imported sea food) and the human body. Kassem et al. (2019) reported that the *mcr-1* gene was present in Lebanese irrigation waters, and most of the isolates were MDR. This observation is worrying because it can affect a wide variety of environments, including the Mediterranean basin.

## 2 Methods

### 2.1 Data collection

The research was performed using the PRISMA-ScR Checklist. Systematic searches were performed in PubMed, Scopus, Sciencedirect, Web of Science, and Researchgate databases in English without limiting the year of publication. We selected articles, book chapters, reviews, reports, short communication and letters to the editor that had published or accepted status. Some publications were selected from the references of the articles found. The mainline of search was colistin-resistant *E. coli* strains (with *mcr-1* gene) found specifically in cow's milk and dairy products, such as yogurt, cottage cheese, cheese, and butter. However, it was apparent from the number of articles that this area is under-researched, so we extended the study to other samples, such as manure, faeces, meat, intestinal tract, and environment. The following keywords were used in the search: „colistin resistance”, „colistin and milk”, „*mcr-1* and milk”, „ESBL (Extended Spectrum Beta-Lactamase) and milk”, „cheese and colistin”, „cheese and *mcr-1*″, „yogurt and *mcr-1*″, „yogurt and colistin”, „*E.coli* and colistin”.

## 3 Results

Since their discovery in 2015, several studies focused on the *mcr* genes, their occurrence and modification. A number of reviews were written on the subject, for example Gharaibeh and Shatnawi (2019) published a great article showing the widespread use of colistin in the world. This review also reports that some research groups are considering new antibiotics or an alternative solution to replace colistin. *mrc* has been found several times in human samples, posing a potential hazard to human health. Hu et al. (2017) examined fecal samples from children and found that five samples were positive for the *mcr-1* gene. The children were 2–27 months old and most of them had not yet received a polymyxin antibiotic; two of them were still breastfeeding and have not yet been started on solid foods. It was also found that *mcr-1*-containing plasmids and other resistance determinants could possibly be co-transferred to other species, which helps the spread of these genes between species. In the Castanheira et al. (2016) study, 390 samples were collected from 183 hospitals, and 19 of these showed colistin resistance (Belgium (1 isolate), Brazil (1 isolate), Germany (5 isolates), Hong Kong (1 isolate), Italy (4 isolates), Malaysia (1 isolate), Poland (1 isolate), Russia (1 isolate), Spain (3 isolates) and the United States (1 isolate)). Al-Tawfiq et al. (2017) reviewed articles from the last 10 years, and found that animal (mainly consumed as food) and human samples were positive for the *mcr-1* gene. Colistin-resistant *E. coli* isolates of human origin were reported from seven countries in Southeast Asia (Cambodia, Indonesia, Laos, Malaysia, Singapore, Thailand, and Vietnam) (Malchione et al., 2019).

The occurrence of the *mcr-1* gene detected in human samples on different continents is shown in Table 1 (Luo et al., 2020).

**Table 1:** The *mcr-1* gene found in humans by continent.

Continent	Number of human samples containing the *mcr-1* gene
Europe	186
America (North and South)	1788
Asia	248
Africa	59
Australia	2

### 3.1 Occurrence of *E.coli* in samples

Oh et al. (2020) investigated the *mrc-1* gene in approximately 1,300 fresh produce (leaves, stems, roots and fruits) in South Korea. The gene was detected in *E. coli* isolates from one lettuce. The same *E. coli* ST10 strain has been detected in several human and animal samples (Manges et al., 2015), which suggests that the antibiotic used in veterinary medicine is excreted and transferred into manure, which is then absorbed by the plants.

Since the *mcr-1* gene is, in most cases, carried by *E. coli*, it is also very important to know how often and in what diseases or in which samples this bacterium occurs. One study (Metzger et al., 2018) examined primiparous Holstein cows with no history of mastitis. The milk sample was taken directly from the gland cistern of 20 cows, as well as looking at the skin of teats and determining the microbiota, where a large number of Gram-negative bacteria were found. All of this may be a cause for concern, as the *mcr-1* gene occurs mainly in Gram-negative bacteria. Odenthal et al. (2016) in Germany took 866 bulk tank milk samples, of which ESBL-producing strains were found in 82 samples. This might pose a problem, because ESBL-producing strains may also carry genes encoding resistance to other antibiotics, and therefore ESBL-producing strains are characterized by MDR.

Ntuli et al. (2016) have shown that *E.coli* strains (and other Enterobacteriaceae) occur not only in raw milk, but also in pasteurized milk, and several of these strains have been shown to be resistant. Sonnier et al. (2018) examined 488 bulk tank milk samples in the US, 30.5% of which was positive for the *E. coli* virulence gene. Dell Orco et al. (2019) characterized a total of 149 *E. coli* isolates from bulk tank milk and raw milk filters, of which 35,6% were pathogenic. They were tested 16 different types of antibiotics and received high MIC in some antibiotic, confirming the potential risk these raw bacteria pose for the spread of antimicrobial resistance in milk.

Unfortunately, colistin resistance has not been looked at by the researchers.

### 3.2 Occurrence of colistin resistance in samples

Monitoring colistin use is a difficult task in several ways. Colistin is not allowed to be used on cattle farms in China, therefore little or no publications were written on the subject (Wang et al., 2020b).

In some countries, such as the U.S.A., Finland, Norway and Iceland, colistin is not permitted for use in food-producing animals as well as polymyxins (European Medicines Agency 2016). Although prohibited in some countries for use in certain animal species, it is illegally available, so for assessing antibiotic use, we need to depend on the honesty of the farm manager. Due to these circumstances, mapping the use of colistin is a rather difficult task. In a study by Ojo et al. (2016), the high levels of antibiotic use among livestock farms in some areas of Nigeria is described. Of the 454 livestock producers, 89 used colistin. Antibiotic resistance in farm animals has been studied in several articles, which show that pigs and poultry have a much higher incidence of colistin resistance genes than cattle (Garch et al., 2018; García et al., 2018). Since there are several farms where not just one type of animal is kept, or there are meat processing plants, where multiple types of animals are slaughtered, the bacterium can easily be transferred from one animal to another, posing a significant problem.

Fu et al. (2018) also detected colistin A and colistin B in 29.63% of feed samples in different provinces of China. The *E. coli* O157: H7 strain, that can lead to fatal disease in people, is very common in beef cattle as was shown by Beauvais et al. (2018). In this research, 2759 fecal samples were examined between 2014 and 2015, and 21.2% of the samples gave a positive result. It would have been worthwhile to look at colistin resistance because it is common in this strain. Magwira et al. (2005) investigated the O157: H7 strain in 400 meat samples, and the *E.coli* from 26% of the samples were resistant to colistin. In another study, the researchers collected milk or fecal samples from cattle after mastitis or enteric infection between 2004 and 2010 in France and Germany. *E.coli* was identified in 150 samples, of which 3 showed MDR, and 3 showed colistin resistance (Brennan et al., 2016).

Irrgang et al. (2016) examined samples coming from cattle and other species in German livestock and food. These samples were obtained between 2010 and 2015 from the German monitoring program on antimicrobial resistance in zoonotic agents. From 909 beef cattle faeces samples, 6 were positive for colistin resistance, 1 resistant strain was found in 196 bulk tank milk samples, and 1 resistant strain was detected in 76 cheese samples.

Tark et al. (2017) in South Korea studied milk samples from 374 cows suffering from mastitis, which samples were collected animal agency and veterinary service agencies between 2012 and 2015. Colistin resistance was detected in 0.5% of isolated *E.coli* strains. The presence of the *mcr-1* gene was not observed in the samples.

### 3.3 Occurrence of *mcr-1* gene

#### 3.3.1 In animal samples

Due to the large amount of antibiotic use in farm animals, which is sometimes unnecessary, the antibiotic content of the meat sold is also high. All this contributes to the development of antibiotic resistance. Many researchers have studied animals or their meat for the presence of colistin-resistant strains, which formed under the influence of antibiotics. Filioussis et al. (2019) studied 400 cows suffering from mastitis on 23 Greek farms. 89 samples were positive for colistin resistance from half of the farms and about 6 samples showed multidrug resistance and contained the *mcr-1* gene.

The use of animal manure in agriculture is quite widespread, so special attention should be paid to its quality. Due to the frequent use of antibiotics, the incidence of antibiotic-resistant strains in manure is high. 51 animal manure samples were examined by Gao et al. (2019), where the *mcr-1* gene was detectable in 31% of the samples. However, all *mcr-1* resistant strains were successfully eliminated by thermophilic composting. Zhang et al. (2019) also studied fecal animal samples in China, but these farms rarely use colistin. From 156 cow samples, 26.92% showed colistin resistance. The *mcr-1* gene was detected in 71.43%, almost all from milking cows, while both *mcr-1* and *mcr-2* genes were detectable in 3 cows. Zheng et al. (2019) examined faecal samples of 651 dairy cows in China in 2016, of which 290 contained ESBL-producing strains, and 3 were positive for the *mcr-1* gene. He et al. (2017) also detected the *mcr-1* gene in one sample from 203 cattle faecal samples. Ngbede et al. (2020) examined 1119 samples from humans, camels, cattle, dogs, pigs and poultry. 583 bacterial strains were identified in these samples, of which 17.0% (99/583) were colistin-resistant. The *mcr-1* gene was also detectable in 1 of the 36 cattle samples. Haenni et al. (2016) collected isolates from the faeces of calves suffering from diarrhea between 2005 and 2014 on French farms and detected the *mcr-1* gene.

In Brazil, *mcr-1* and other genes responsible for colistin resistance were isolated from healthy bovine faecal samples. This is also a major concern, because Brazil is one of the largest exporters of beef (Palmeira et al., 2018).

Between 2006 and 2014, colon or caecal isolates were collected from several European countries by Garch et al. (2018) from all major food-producing species (beef cattle, slaughter pigs and broiler chickens). Fortunately, none of the cattle samples were found to be *mcr-1* positive.

In South-Korea, Belaynehe et al. (2018) examined 636 healthy animal faecal samples between 2014 and 2017, of which 341 were derived from cattle. A colistin-resistant strain was not detected in these samples either.

#### 3.3.2 In food

In the U.S., Wang et al. (2020a) examined 5,169 samples, of which 1,057 were beef and 1,369 were minced beef (the other samples were chicken, catfish, pork, and poultry). Strains with the *mcr-1* gene were not detected. However, the *mcr-1* gene has been detected in beef in Canada (Mulvey et al., 2016), and in Gram-negative bacteria isolated from ready-to-eat food in Bolivia (Sennati et al., 2017). A review by Oliveira et al. (2019) summarized results from clinical, animal, food, and environmental samples examined in Latin America. Of the 18705 isolates, 550 contained some type of *mcr* gene, 95.8% of which was *E. coli* (some from cattle). Latin America uses large amounts of colistin, which is a huge problem in terms of Brazil's significant meat exports (Silva et al., 2013).

Chen et al. (2017) collected 1371 food samples in China, these included meat products (chicken, duck, pork, beef and mutton), seafood (shrimp, fish and shell fish), dairy products (yogurt, milk cheese and butter), fresh produce (lettuce, cabbage, broccoli, cauliflower etc.) and other food products (tofu etc.), and 36% were positive for the *mcr-1* gene (some from beef).

As EFSA reports show, colistin resistance was also detected in 0.9% of calves under one year of age and in bovine meat (EFSA 2015; 2017).

Table 2 summarizes the prevalence of the *mcr-1* gene detected in cattle and beef by country.

**Table 2:** Occurrence of the *mcr-1* gene in bovine samples.

Country	Sample	Number of samples positive for *mcr-1*	Ratio to total samples	Reference
Algeria	bovine manure	1	12,5%	Touati et al., (2020)
Belgium	cattle	1	0,03%	Garch et al., (2017)
Belgium	calves	6	11,5%	Xavier et al., (2016), Kumar et al., (2016)
Canada	lean ground beef	2	0,125%	Mulvey et al., (2016)
China	cattle manure	2	9,52%	Gao et al., (2019)
China	beef	1	3,03%	Sun et al., (2021)
China	beef	10	28,5%	Chen et al., (2017)
China	faecal swabs (cattle)	42	27%	Zhang et al., (2019)
Egypt	beef (raw, ready to eat)	4	3,8%	Sabala et al., (2020)
Egypt	beef sausage	1	0,7%	Sadek et al., (2020)
France	calves	4	0,4%	Gay et al., (2019)
France	cattle	9	0,28%	Garch et al., (2017)
France	fecal (cattle)	1	0,66%	Brennan et al., (2016)
Germany	veal, calves, faeces	22	2,45%	Irrgang et al., (2016)
Germany	calves, colon content	4	0,83%	Irrgang et al., (2016)
Germany	veal	1	1,42%	Irrgang et al., (2016)
Italy	cattle	2	0,09%	Garch et al., (2017)
Japan	cattle (mastitis)	4	0,042%	Suzuki et al., (2016)
Netherland	veal calves	2	13,3%	Veldman et al., (2016)
Nigeria	cattle, rectal swab	1	2,77%	Ngbede et al., (2020)
Spain	healthy cattle younger than 1 year	6	0,94%	Hernández et al., (2017)
Spain	cattle faeces	5	3,28%	Hernández et al., (2017)
Spain	with waste milk feeded to calves (faecal sample)	31	8%	Maynou et al., (2017)
Taiwan	beef meat	1	1,12%	Kuo et al., (2016)
Tunisia	bovine faeces	3	2,5%	Hassen et al., (2019)
Portugal	meat and animal sample, bovine	0/364	0%	Clemente et al., (2019)
South America	cattle	0/158	0%, but 13,9% colistin resistant	Fernandes et al., (2016)
Europe	cattle	0/3101	0% but 0,72% colistin resistant	Garch et al., (2018)

From the table, we can see that the *mcr*-1 gene is found in almost every continent in some of the bovine samples. In the last 3 items of the table, colistin-resistant strains were detected in the tested samples, without *mcr*-1 gene.

The presence of the *mcr-1* gene in bovine samples were also reported by four research groups, although colistin is not allowed to be used in these animals in China. Although the strain with the fewest *mcr-1* gene was found in cattle among meat-producing animals, it is a matter of concern that such a strain was also found on four continents. Bovine faecal samples where resistant strains have been detected are problematic, because the manure is used for fertilization in agriculture in many countries. This presents a potential danger through the transmission of resistance to plants. Table 3 summarizes the number of strains with the *mcr-1* gene detected in cow's milk and dairy products.

**Table 3:** Colistin resistance can be detected in dairy samples from cattle by country.

Country	Sample	Number of strains with *mcr-1*	Ratio to total samples	Reference
China	mastitic milk	5	1%	Liu et al. (2020a)
China	bovine mastitic milk	5	0,245%	Liu et al., (2020b)
Egypt	ready to eat karish cheese	4	2%	Hammad et al., (2019)
Greece	single-quarter mastitic milk samples	6	1,5%	Filioussis (2019)
South Korea	bovine mastitic milk	2	0,5%	Singh et al., (2015)
Tunisia	bovine raw milk	1	1%	Hassen et al., (2019)
France	milk, cheese	107, just colistin resistance	62%	Coton et al., (2012)
India	bovine milk	0/182	0%, but 6,5% ESBL-positive	Batabyal et al., (2018)
Nigeria	“nono” fermented cow milk	16 just colistin resistant	32%	Shehu and Adesiyun (1990)

**Figure 1:**
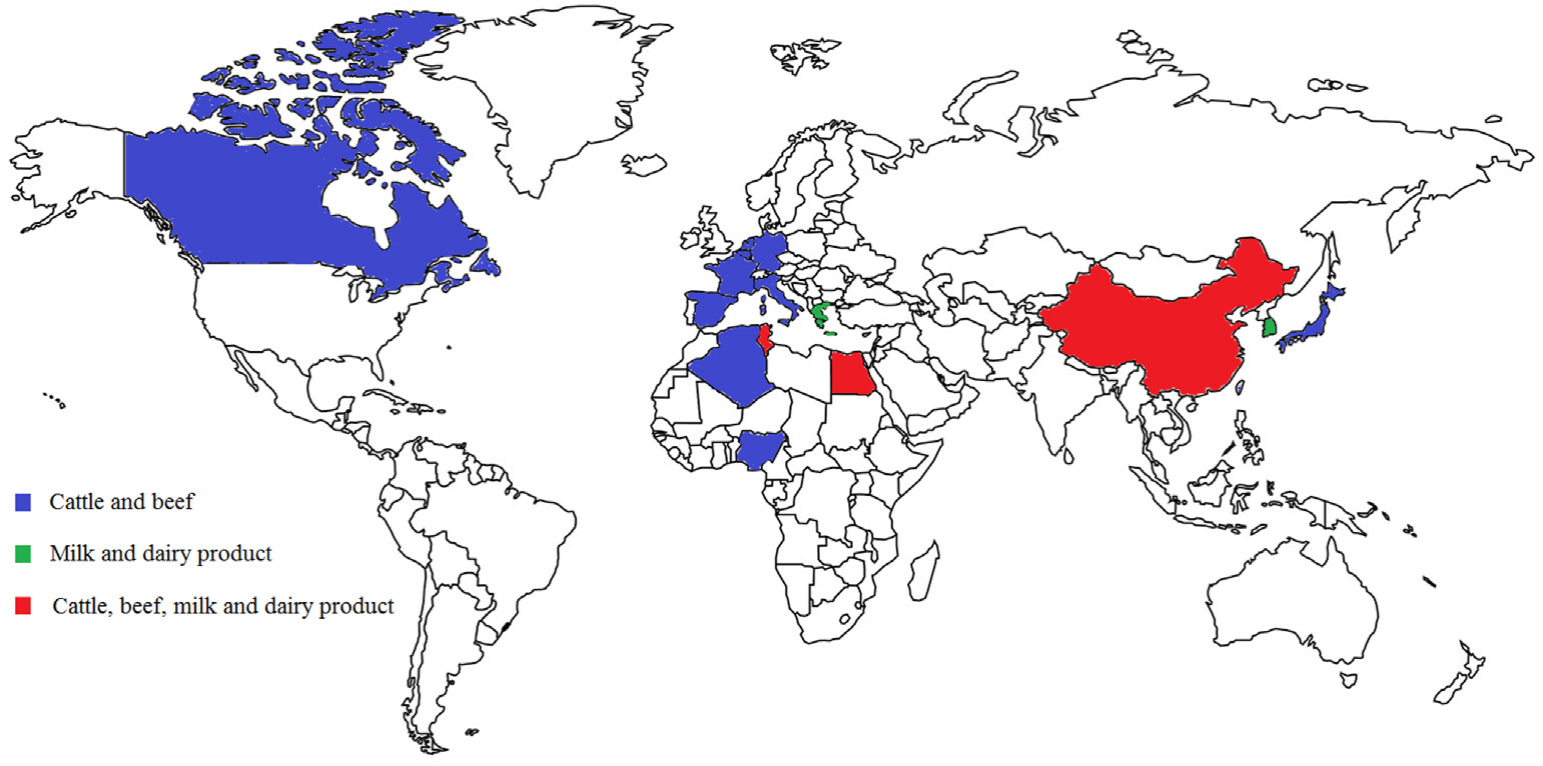
The global detection of the *mcr-1* gene in milk dairy products and cattle.

A total of 9 articles were found which reported that the *mcr-1* gene, or colistin-resistant strain, was isolated from milk and dairy products. Several articles report resistance of colistin in dairy product, but the *mcr* gene not detected. However, no comprehensive study has been carried out in the EU or in countries like India, where milk consumption is significant. We can see that although the prevalence of strains containing the *mcr-1* gene is small (Figure 1), it is unfortunately also found in countries where colistin use is minimal.

Three strains with the *mcr-1* gene was isolated in samples from a wastewater treatment plant in Germany by Schages et al. (2020), and several colistin-resistant strains were also found. Lekunberri et al. (2017) examined raw and treated samples from wastewater treatment plants, and found that treatment reduces the number of colistin-resistant strains, although it does not completely kill them. Furthermore, it was also found that the presence of the *mcr-1* gene has increased over the years. In several countries, municipal wastewater is used for irrigation purposes in agriculture (Carter et al., 2020), therefore significant problems might arise due to gene transfer in plants.

The *mcr-1* gene was not detected in milk and dairy products in a large-scale study in the US (Wang et al., 2020a). However, it has not been studied in many countries to this date, so we have little information on the presence of *mcr-1* genes in milk and dairy products worldwide, even though consumption of milk and dairy products is significant. Since colistin resistance has already been shown in probiotics (Li et al., 2020), which are favorably used in the manufacture of dairy products, it would certainly be worthwhile to carry out a comprehensive study regarding *mrc-1* in milk.

## 4 Conclusions

AMR is a growing problem in the world. Use of "last-resort" antibiotics is especially risky if resistance develops against them. Because the use of colistin is rarely allowed in dairy farms, the presence of *mcr* genes in milk has been studied in quite a few countries. However research is important, as resistance genes can also be transmitted to cattle by horizontal gene transfer and from the environment.

To get an accurate picture of the prevalence of AMR, it would be advisable to monitor milks, especially in countries where antibiotic use is significant or where such measurements have not yet been made. This would give an accurate picture of the presence of *mcr* genes worldwide.

In order for last-resort antibiotics to remain effective in human medicine, their use in animal husbandry should be discontinued. Instead, the simultaneous use of multiple antibiotics at higher doses in diseased animals may be effective. In addition, efforts should be made to strengthen the immune system of the animals by adding pre- or probiotics to the feed.

Antibiotics getting wastewater treatment plants cannot be properly removed, so they contribute to the development of resistant microbes when they enter the environment. Wastewater treatment plants need to be upgraded to reduce the spread of antibiotic resistance.

The use of these antibiotics must be limited in order to ensure that they remain effective in hospital and veterinary treatments, especially in non-targeted applications.

## Declarations

### Author contribution statement

All authors listed have significantly contributed to the development and the writing of this article.

### Funding statement

This work was supported by the 10.13039/501100000780European Union and co-financed by the 10.13039/501100004895European Social Fund (EFOP-3.6.1-16-2016-00024).

### Data availability statement

Data included in article/supplementary material/referenced in article.

### Declaration of interests statement

The authors declare no conflict of interest.

### Additional information

No additional information is available for this paper.
